# Optimizing the Egli Model for Vehicular Ultra-Shortwave Communication Using High-Resolution Remote Sensing Satellite Imagery

**DOI:** 10.3390/s25175242

**Published:** 2025-08-23

**Authors:** Guangshuo Zhang, Peng Chen, Fulin Wu, Yangzhen Qin, Qi Xu, Tianao Li, Shiwei Zhang, Hongmin Lu

**Affiliations:** 1 School of Electronic Engineering, Xidian University, Xi’an 710071, China; zhangguangshuoemc@stu.xidian.edu.cn (G.Z.); 1702310030@stu.xidian.edu.cn (P.C.); fulinwu@stu.xidian.edu.cn (F.W.); 22021110257@stu.xidian.edu.cn (Y.Q.); 23021211636@stu.xidian.edu.cn (Q.X.); 23021211957@stu.xidian.edu.cn (T.L.); 2 China North Vehicle Research Institute, Beijing 100072, China; tt2nd@126.com

**Keywords:** high-resolution remote sensing satellite imagery, radio wave propagation loss, vehicle, ultra-shortwave communication, model optimization

## Abstract

The traditional radio wave propagation models exhibit several limitations when they are employed to predict the path loss for vehicular ultra-shortwave wireless communication. To addresses these challenges, an optimized approach for Egli model based on the high-resolution remote sensing satellite image is proposed in this study. The optimization process includes three components. First, a method for calculating the actual equivalent antenna height is introduced, utilizing high-precision remote sensing satellite imagery to obtain communication path profiles. This method accounts for the antenna’s physical length, vehicular height, and local terrain characteristics, thereby providing an accurate representation of the antenna’s effective height within its operational environment. Second, an equivalent substitution method for ground loss is developed, utilizing surface information derived from high-precision remote sensing satellite images. This method integrates ground loss directly into the Egli model’s calculation process, eliminating the need for separate computations and simplifying the model. Third, leveraging the Egli model as a foundation, the least squares method (LSM) is employed to fit the relief height, ensuring the model meets the requirements for ultra-short wave communication distances under line-of-sight (LOS) conditions and enhances suitability for real-world vehicular communication systems. Finally, the validity and accuracy of the optimization model are verified by comparing the measured data with the theoretical calculated values. Compared with the Egli model, the Egli model with additional correction factors, and the measured data, the average error of the optimized model is reduced by 8.98%, 2.09%, and the average error is 0.45%.

## 1. Introduction

As a crucial component of modern wireless communication technology, vehicular ultra-shortwave communication has found extensive applications in military, public security, emergency rescue, and other critical fields [1,2,3,4,5,6]. Operating within the frequency band of 30 MHz to 300 MHz (very high frequency, VHF), this technology offers several advantages, including low propagation loss, strong diffraction capability, and excellent anti-interference performance, making it particularly suitable for communication in complex terrains and mobile environments. In recent years, with the rapid advancement of special vehicle technologies and intelligent transportation systems, there has been an increasing demand for higher reliability and practical performance in vehicular communication systems [7,8,9,10,11,12,13,14]. A key research focus in this domain has been the accurate prediction of radio wave propagation loss and the optimization of communication link performance, especially within line-of-sight (LOS) communication ranges [15,16,17,18,19,20].

In the context of diverse propagation scenarios, most loss report methodologies focus on fine-tuning propagation loss models. Previous studies have employed various optimization techniques: genetic algorithms for path loss model optimization [21] and particle swarm optimization demonstrating superior performance over genetic algorithms for Hata model tuning [22]. Additionally, the least-square approximation method has been widely adopted for path loss model optimization due to its simplicity and effectiveness in error minimization [23,24,25,26]. However, practical applications reveal that traditional radio wave propagation models (e.g., the Egli model and Okumura–Hata model) show limited accuracy in vehicular communication scenarios [27,28,29,30]. This limitation primarily stems from their inadequate consideration of critical factors such as terrain relief height, vehicle-mounted antenna heights, and surface loss [31,32,33,34]. Notably, while numerous tuning methods have been proposed, the Egli model has received comparatively less attention in the literature. The fundamental objective of all these optimization techniques remains consistent: to minimize prediction errors associated with propagation models. This underscores the significant engineering importance of developing high-precision radio wave propagation models specifically designed for ultra-shortwave vehicular communications [35,36,37,38,39,40]. Current propagation models applied to vehicular systems exhibit several critical limitations:

(1) The operational frequency range of vehicular communication systems fails to align with the frequency specifications of conventional radio wave propagation loss models.
(2) The vehicle-mounted antenna height configuration falls outside the optimal range defined by current radio wave propagation loss models.
(3) The models lack comprehensive consideration of the terrain relief height influences on radio wave propagation loss.

In recent years, the rapid development of remote sensing technology has made the acquisition of remote sensing data more accessible, particularly high-resolution remote sensing images. High resolution remote sensing data enable city modeling for signal propagation analysis [41,42,43,44,45,46,47], with methods like SBL-based REM [42,44,47] offering ray-tracing capabilities. The high-resolution images support detailed urban modeling required for realistic propagation simulation, particularly for capturing urban structure effects like signal attenuation and diffraction losses in radio wave propagation model optimization. The application of high-resolution satellite remote sensing image to model optimization for vehicular communication systems has significant advantages, including improved accuracy and enhanced realism.

To address these limitations in the vehicular communication systems, an optimized approach to the Egli radio wave propagation model based on high-resolution satellite remote sensing imagery is proposed in this paper. The main contributions of this article are as follows:

(1) Taking into account the altitude profile derived from high-precision remote sensing satellite images, along with the height of the vehicle and the physical length of the antenna, a method for calculating the actual equivalent antenna height is proposed. This method ensures a more accurate representation of the antenna’s effective height in relation to its environment.
(2) Based on surface information derived from high-precision remote sensing satellite images and combined with the actual equivalent height of vehicle-mounted antennas an equivalent substitution method for surface loss is introduced. This method allows surface loss to be seamlessly integrated into the Egli model’s calculation process, eliminating the need for separate computations and thereby simplifying the overall model.
(3) Leveraging the Egli model as a foundation, the least squares method (LSM) is employed to fit the terrain relief height. This fitting ensures that the model meets the requirements for ultra-short wave communication distances under LOS conditions.

The remainder of this paper is organized as follows. Section 2 presents the theoretical foundation, including (1) justification for selecting the Egli model and (2) the principles and advantages of applying LSM optimization to the Egli model. Section 3 details the three core optimization components: (1) computation of equivalent antenna height, (2) surface loss equivalent substitution, and (3) terrain relief height fitting. Section 4 validates the optimized model’s accuracy through field tests with vehicular communications and provides performance analysis. Finally, Section 5 concludes with key findings and their practical implications.

## 2. Model and Theoretical Basis

### 2.1. Model Selection

The selection of an appropriate propagation model is contingent upon meeting several critical technical specifications and operational requirements:

(1) The selected model must demonstrate effective radio wave propagation capabilities within the very high frequency (VHF) spectrum, particularly operating in the specified frequency range of 30–300 MHz, which corresponds to the ultra-shortwave communication band. Table 1 lists the operating frequency range of the classical radio wave propagation models.

(2) The selected model should meet the requirements for LOS communication over distances of 15–30 km. Table 2 lists the propagation distance range of the classical radio wave propagation models.

(3) The selected model must be compatible with vehicle-mounted antenna height configurations (typically 10–15 m above ground level) to ensure operational suitability in real-world communication scenarios. Table 3 lists the antenna height range of the classic radio wave propagation models.

(4) The selected model must account for moderately undulating irregular terrain, including hills, valleys, and urban environments. Table 4 lists the terrain requirements of the classical radio wave propagation models.

The selection of the Egli model for optimization is selected through analysis of vehicular ultra-shortwave communication needs and systematic comparison of existing propagation models.

The Egli model (1957) [49], or “Hill Terrain Propagation Model with 50-Foot Average Slope Height,” is an empirical radio propagation model for irregular terrain. It predicts path loss for Frequency: 40–400 MHz (extendable to 1 GHz) and Distance: 0–64 km.

The Egli model’s radio wave propagation loss L is given in Equation (1).

(1) $$L=88+20\mathrm{lg}(f)-20\mathrm{lg}\left(h_{1}h_{2}\right)+40\mathrm{lg}(d)+K_{h}$$

where f is the radio wave frequency (MHz); $h_{1}$ and $h_{2}$ are the transmitting and receiving antenna heights (m); d is the distance between the transmitting and receiving antenna (km); and $K_{h}$ is the terrain correction factor.

The terrain correction factor $K_{h}$ varies with radio frequency ranges, as specified in Equations (2)–(4).

(2) $$K_{h}=1.667-0.1094\Delta h;\;25\;\mathrm{MHz}<f<150\;\mathrm{MHz}$$

(3) $$K_{h}=2.25-0.1476\Delta h;\;150\;\mathrm{MHz}<f<162\;\mathrm{MHz}$$

(4) $$K_{h}=3.75-0.2461\Delta h;\;450\;\mathrm{MHz}<f<470\;\mathrm{MHz}$$

where Δh is the terrain relief height (m).

When Δh≤15, $K_{h}=0$; when Δh>15, $K_{h}$ needs to be corrected.

### 2.2. Least Squares Method

The least squares method (LSM) is a mathematical optimization technique that determines the optimal function fitting a given dataset by minimizing the sum of squared residuals [53]. The exponential least squares model represents a nonlinear regression approach specifically designed to characterize data exhibiting exponential growth or decay patterns with respect to independent variables. Its general form is expressed as [54]:

(5) $$y=a\cdot e^{bx}$$

where y is the dependent variable; x is the independent variable; and a and b are the parameters to be evaluated.

Due to the nonlinear nature of the exponential model, direct determination of parameters a and b using the LSM presents computational challenges. Typically, this problem can be addressed through linearization by applying the following transformation steps.

(6) ln(y)=ln(a)+bx

Let Y=ln(y) and A=ln(a), then Equation (6) is rewritten as Equation (7).

(7) Y=A+bx

Following the least squares principle, we obtain the system of equations in Equation (8) by setting the partial derivatives with respect to A and b equal to zero.

(8) $$\left\{\begin{array}{c} nA+b\sum x=\sum Y\\ A\sum x+b\sum x^{2}=\sum xY \end{array}\right.$$

where n is the number of data points.

Equations (9)–(11) are obtained by solving Equation (8).

(9) $$b=\frac{n\sum xY-\sum x\sum Y}{n\sum x^{2}-{\left(\sum x\right)}^{2}}$$

(10) $$A=\frac{\sum Y-b\sum x}{n}$$

(11) $$a=e^{A}$$

## 3. Optimization Process

The key process includes three components: (1) vehicle antenna effective height calculation, (2) surface loss equivalent substitution, and (3) terrain relief height fitting optimization. Each component is detailed below.

### 3.1. Actual Equivalent Antenna Height Calculation

**Figure 1:**
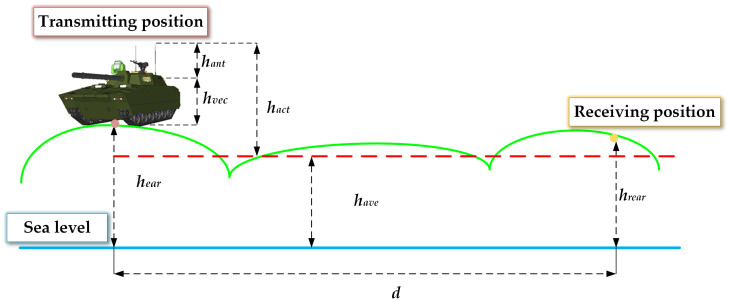
Illustration of actual equivalent antenna height.

Since vehicle-mounted antennas are significantly lower than base station antennas and vehicle height cannot be neglected, the effective antenna height becomes a critical parameter for model optimization, directly affecting the model’s accuracy. Figure 1 shows the illustration of actual equivalent antenna height.

(12) $$h_{act}=h_{ant\;}+h_{vec\;}+h_{ear\;}-h_{ave}$$

where $h_{act}$ is the actual equivalent height of vehicle-mounted antenna (m); $h_{vec}$ is the vehicular height (m); $h_{ant}$ is the physical length of vehicle-mounted antenna (m); $h_{ear}$ is the altitude at which the vehicle is located; and $h_{ave}$ is the average altitude of the actual environment of vehicle communication.

### 3.2. Surface Loss Equivalent Substitution

In ground communication systems, surface loss is a fundamental component of radio wave propagation model optimization that cannot be ignored. The reasons are as follows:

(1) Energy absorption by the ground: when a vehicle engages in wireless communication on the ground, the ground absorbs a portion of the radio wave energy during propagation, leading to signal attenuation. The extent of energy absorption varies depending on the ground type, such as soil, water, vegetation, or urban structures.
(2) Terrain irregularities: irregular terrain features such as hills, valleys, and urban structures can scatter, reflect, and diffract radio waves, distorting their propagation path and reducing signal strength.
(3) Frequency dependency: the surface loss is related to the working frequency. In the VHF band, the interaction between radio waves and the ground becomes more pronounced, resulting in greater energy loss.
(4) Impact on communication distance: in vehicular communication systems, the proximity of both the transmitter and receiver to the ground leads to significant surface loss, which markedly diminishes signal strength and directly impacts the system’s effective communication distance.

The operational frequency range of vehicular ultra-shortwave communication radios typically spans from 30 MHz to 88 MHz. In this frequency band, as the height of the vehicle antenna decreases, the contribution of ground waves becomes increasingly significant. When the antenna height is reduced to a certain critical value, known as the minimum effective antenna height $h_{0}$, surface losses can no longer be neglected. Vehicular communication systems are typically equipped with vertically polarized whip antennas. The minimum effective antenna height $h_{0}$ of such an antenna can be determined using Equation (13).

(13) $$h_{0}=\frac{\lambda {\left[{\left(\varepsilon +1\right)}^{2}+{\left(60\sigma \lambda \right)}^{2}\right]}^{1/4}}{2\pi }$$

where ε is the ground’s relative permittivity; σ is the ground’s conductivity (S/m); and λ is the radio wave length (m).

The equivalent antenna height $h_{\mathrm{eff}}$ is obtained from Equation (14). Substituting $h_{\mathrm{eff}}$ into the Egli model eliminates separate surface loss calculations. This optimization simplifies the model’s computation while maintaining accuracy.

(14) $$h_{\mathrm{eff}\;}=\sqrt{h_{0}+h_{act}}$$

where $h_{act}$ is the actual equivalent antenna height (m).

### 3.3. Terrain Relief Height Fitting

As described in Section 2.1, the Egli model adjusts Δh based on empirical curves derived from extensive measurements [49], establishing the functional relationship between Δh and d in Equation (15).

(15) $$\Delta h(d)=\Delta h\left(1-\frac{0.8}{e^{0.02d}}\right)$$

Equation (15) establishes a positive correlation between Δh and d. As communication distance increases, Δh(d) approaches an asymptotic limit. When Δh(d) reaches 90% of Δh’s asymptotic value (Equation (16)), the propagation distance d can be derived (Equation (17)).

(16) $$1-\frac{0.8}{e^{0.02d}}=90\%$$

(17) d=50ln8≈103.97(km)

The maximum LOS communication distance for vehicle VHF radio is 30 km—only one-third of the theoretical value calculated in Equation (17). This significant discrepancy in tactical wireless communication range is addressed by incorporating Section 3.1 and Section 3.2.

(18) $$\Delta h^{\prime }(d)=\Delta h\left(1-\frac{0.14}{e^{0.01d}}\right)$$

When $\Delta h^{\prime }(d)$ reaches 90% of Δh’s asymptotic value (Equation (19)), the propagation distance d can be calculated using Equation (20).

(19) $$1-\frac{0.14}{e^{0.01d}}=0.9$$

(20) d≈32(km)

Equation (20) shows that the propagation distance d calculated using the least-squares-fitted Equation (18) satisfies vehicular VHF radio communication distance requirements.

### 3.4. Optimized Egli Model

**Figure 2:**
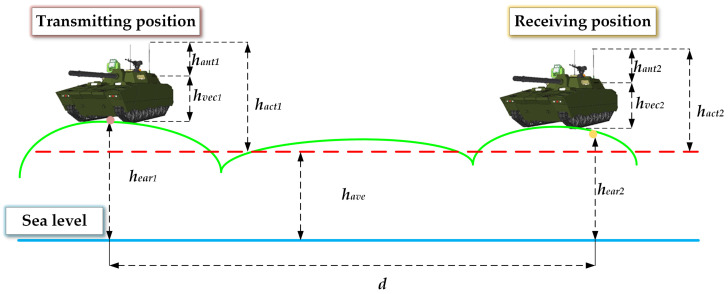
The vehicle-to-vehicle (VTV) communication scenario.

By incorporating the actual equivalent antenna height, surface loss, and fitted terrain relief height into the Egli model, an optimized version of the model is obtained as shown in Equation (21). Figure 2 illustrates the vehicle-to-vehicle (VTV) communication scenario.

(21) $$\begin{array}{cc} L & =88-10\mathrm{lg}\left(\frac{\lambda {\left[{\left(\varepsilon +1\right)}^{2}+{\left(60\sigma \lambda \right)}^{2}\right]}^{1/4}}{2\pi }+h_{ant1\;}+h_{vec1\;}+h_{ear1\;}-h_{ave}\right)\\ & -10\mathrm{lg}\left(\frac{\lambda {\left[{\left(\varepsilon +1\right)}^{2}+{\left(60\sigma \lambda \right)}^{2}\right]}^{1/4}}{2\pi }+h_{ant2\;}+h_{vec2\;}+h_{ear2\;}-h_{ave}\right)\\ & +40\mathrm{lg}(d)+1.667-\left(1-\frac{0.14}{e^{0.01d}}\right)\Delta h+20\mathrm{lg}(f) \end{array}$$

where $h_{ant1}$ and $h_{ant2}$ are the physical heights (m) of antennas mounted on Vehicle 1 and Vehicle 2, respectively; $h_{vec1}$ and $h_{vec2\;}$ denote the vehicle body heights (m) of Vehicle 1 and Vehicle 2; and $h_{ear1}$ and $h_{ear2}$ indicate the terrain altitudes (m) at the locations of Vehicle 1 and Vehicle 2.

## 4. Optimized Model Verification and Analysis

### 4.1. Communication Path Modeling

**Figure 3:**
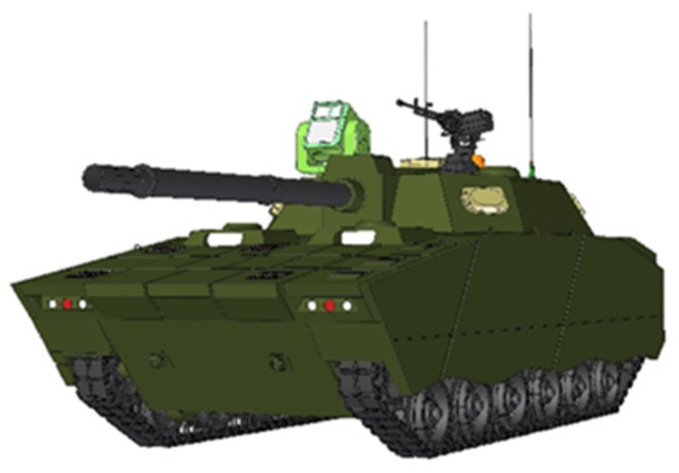
Vehicle 3D model.

The communication experimental subject of this study is a specialized vehicle (Figure 3). Two such vehicles, labeled VEC 1 and VEC 2, are used in the experiments. VEC 1 serves as transmitting ends and VEC 2 acts as the receiving end.

**Figure 4:**
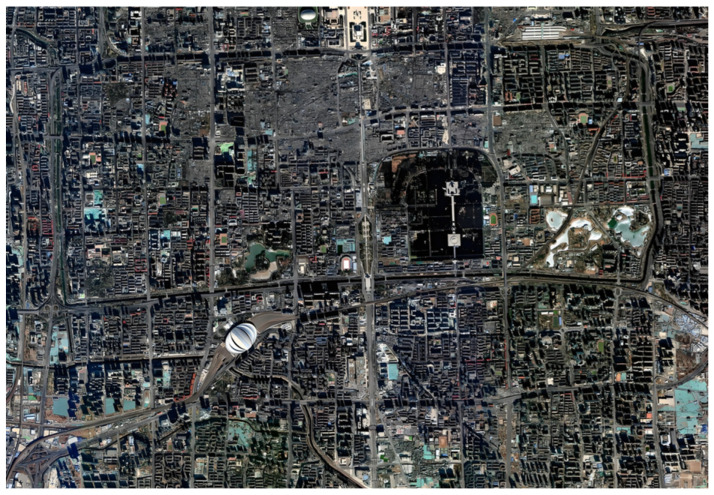
Satellite remote sensing image of the urban area.

**Figure 5:**
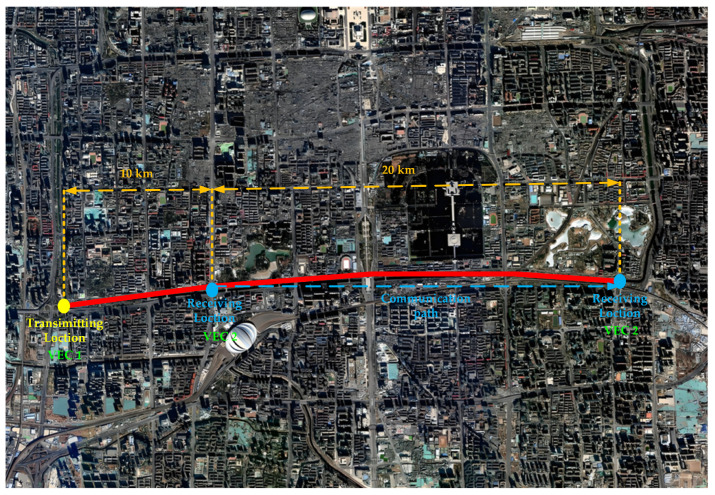
Communication path.

The experimental scene is in a specific urban area of Beijing. Figure 4 shows the satellite remote sensing image of the urban area, with an effective resolution of 5 m after fusion processing. Figure 5 shows the communication path.

**Figure 6:**
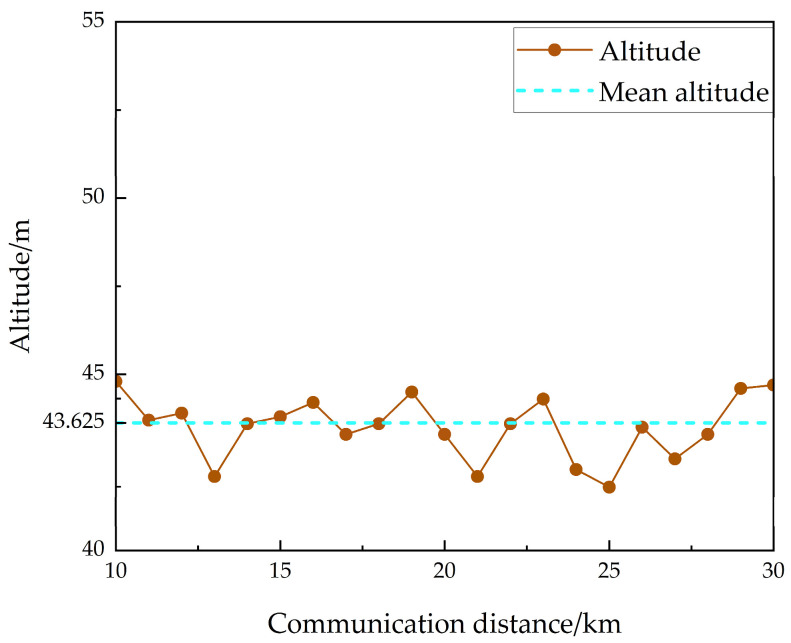
Altitude profile along the communication path.

As shown in Figure 6, the altitude profile along the communication path can be accurately derived from satellite remote sensing imagery. In urban environments, the maximum elevation variation is below 90 m, while the communication distance between the two vehicles ranges from 10 km to 30 km, satisfying the LOS propagation conditions.

This provides essential data for calculating the actual antenna height. Additionally, key propagation parameters along the communication path (the ground’s relative permittivity and conductivity) can be determined, as shown in Table 5. This enables accurate surface loss modeling through equivalent substitution.

### 4.2. Experimental Validation

**Figure 7:**
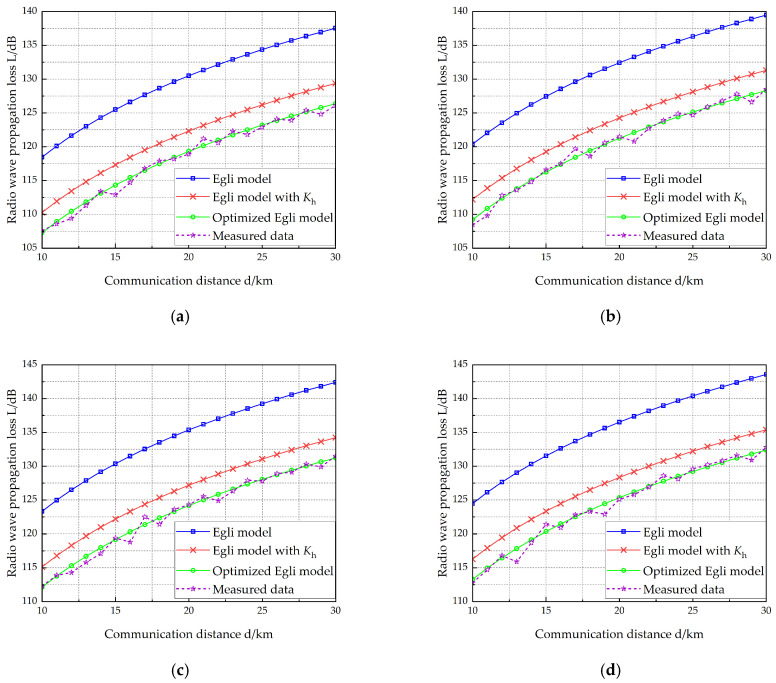
Radio wave propagation loss ($h_{ant1\;}=10\;m$, $h_{ant2\;}=12\;m$). (**a**) *f* = 40 MHz, (**b**) *f* = 50 MHz, (**c**) *f* = 70 MHz, (**d**) *f* = 80 MHz.

**Figure 8:**
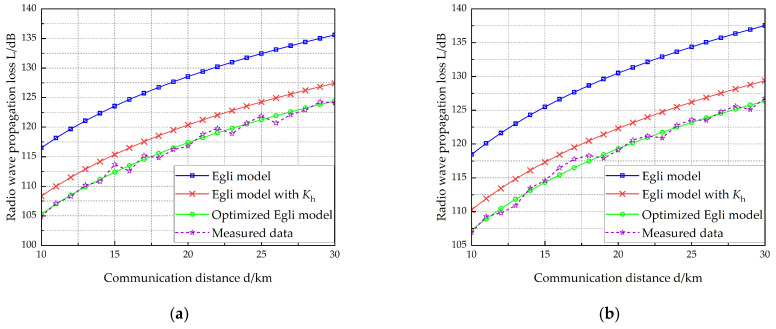

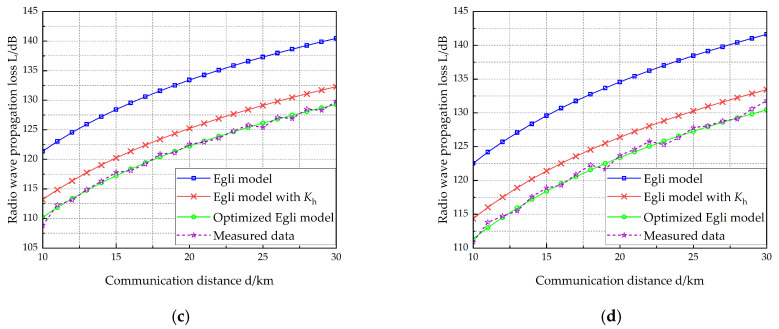
Radio wave propagation loss ($h_{ant1\;}=10\;m$, $h_{ant2\;}=15\;m$). (**a**) *f* = 40 MHz, (**b**) *f* = 50 MHz, (**c**) *f* = 70 MHz, (**d**) *f* = 80 MHz.

**Figure 9:**
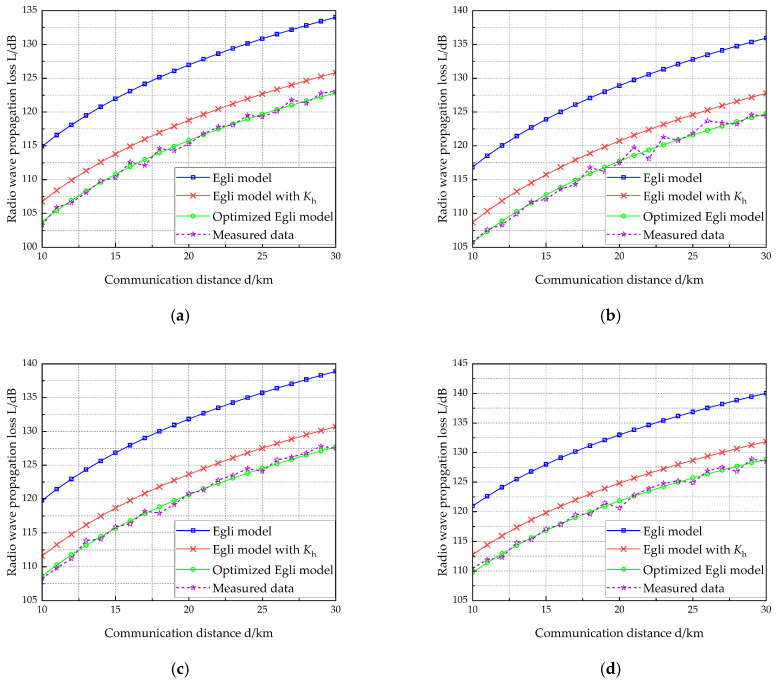
Radio wave propagation loss ($h_{ant1\;}=15\;m$, $h_{ant2\;}=12\;m$). (**a**) *f* = 40 MHz, (**b**) *f* = 50 MHz, (**c**) *f* = 70 MHz, (**d**) *f* = 80 MHz.

**Figure 10:**
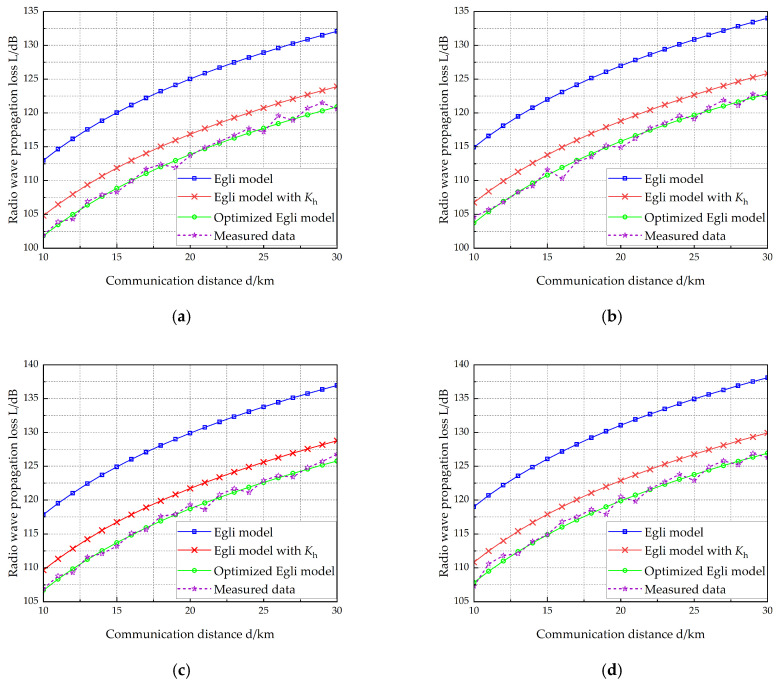
Radio wave propagation loss ($h_{ant1\;}=15\;m$, $h_{ant2\;}=15\;m$). (**a**) *f* = 40 MHz, (**b**) *f* = 50 MHz, (**c**) *f* = 70 MHz, (**d**) *f* = 80 MHz.

VEC 1 is equipped with two antennas measuring 10 m and 15 m in length, respectively, while VEC 2 carries two antennas with lengths of 12 m and 15 m. Table 6 lists the vehicle-mounted radio and antenna parameters of the two vehicles. The working frequency of the radio on VEC 1 is selected at four different points: 40 MHz, 50 MHz, 70 MHz, and 80 MHz. Figure 7, Figure 8, Figure 9 and Figure 10 show the radio wave propagation loss under various conditions.

### 4.3. Result Analysis

The Mean Relative Error (MRE), defined in Equation (22), quantifies the deviation between calculated and measured values. To evaluate the accuracy of the optimized model, this chapter employs MRE as the key metric for assessing discrepancies between model predictions and field measurements.

(22) $$\mathrm{MRE}=\frac{1}{n}\overset{n}{\underset{i=1}{\sum }}|\frac{y_{i}-{\hat{y}}_{i}}{y_{i}}|\times 100\%$$

where $y_{i}$ denotes the i-th measured value; ${\hat{y}}_{i}$ represents the i-th theoretically calculated value; and n is the total number of samples

Using Equation (22), we compute the MRE for three models: (1) the Egli model, (2) the terrain-corrected Egli model, and (3) the optimized model. Table 7 presents the calculated MRE values for each model, along with the reduction in MRE achieved by the improved model over both the original and terrain-corrected Egli models. Table 7 demonstrates that:

(1) The Egli model exhibits an average relative error of 9.43% compared to measured data.
(2) With terrain correction factors, the modified Egli model reduces average relative error to 2.54%.
(3) The optimized model demonstrates significantly improved accuracy, with an average relative error of only 0.45%.
(4) Compared to the Egli model, the optimized version achieves an 8.98% reduction in average relative error.
(5) Compared to the terrain-corrected Egli model, the optimization yields a further 2.09% improvement in accuracy.

### 4.4. Model Analysis

Analysis of the results confirms the effectiveness of the optimized Egli model, demonstrating its improved accuracy for radio wave propagation loss prediction in specialized vehicular scenarios. The model is particularly suitable for applications meeting the following conditions:

(1) Frequency range: VHF band (30–88 MHz);
(2) Communication mode: LOS propagation (≤30 km range);
(3) Terrain type: Gently undulating landscapes (relief height ≤ 90 m).

## 5. Conclusions

An integrated approach to enhance the accuracy and practicality of propagation modeling for vehicular communication systems is proposed in this study. First, we introduce a method to determine the actual equivalent antenna height by combining high-resolution remote sensing satellite data, vehicle height, and antenna dimensions, ensuring precise elevation representation. Second, we develop an equivalent substitution method for surface loss, integrating terrain data derived from remote sensing with the calculated equivalent antenna height. This method directly incorporates surface loss into the Egli model, eliminating standalone computations and simplifying the modeling process. Finally, using the Egli model as a framework, we optimize terrain relief height fitting via the LSM for ultra-shortwave LOS communication. Experimental validation demonstrates a 2.09% improvement in accuracy compared to the terrain-corrected Egli model. Collectively, these contributions advance terrain-aware radio propagation prediction while preserving computational efficiency.

## Figures and Tables

**Table 1:** The operating frequency range of the classical radio wave propagation models.

Model	Free Space Model [48]	ITU-R P.1546 Model [49]	Egli Model [50]	Longley–Rice Model [51]	Okumura–Hata Model [52,53]
Operating frequency range	≤300 GHz	30 MHz–3 GHz	40–400 MHz (extendable to 1 GHz)	20 MHz–40 GHz	150–1500 MHz

**Table 2:** The propagation distance range of the classical radio wave propagation models.

Model	Free Space Model [48]	ITU-R P.1546 [49]	Egli Model [50]	Longley–Rice Model [51]	Okumura–Hata Model [52,53]
Propagation distance range	Unlimited (actually limited by the transmission power)	1–1000 km (land: <300 km)	1–64 km	1–2000 km	>1 km

**Table 3:** The antenna height range of the classic radio wave propagation models.

Model	Free Space Model [48]	ITU-R P.1546 [49]	Egli Model [50]	Longley–Rice Model [51]	Okumura–Hata Model [52,53]
Antenna height range	No strict constraint	Base station: 10–1000 m	Base station: 50–150 m Mobile terminal: 1–10 m	Transmitting end: 10–3000 km Receiving end: (1) base station: 1–500 m (2) mobile terminal: 1–30 m	Base station: 30–200 m Mobile terminal: 1–10 m

**Table 4:** The terrain requirements of the classical radio wave propagation models.

Model	Free Space Model [48]	ITU-R P.1546 [49]	Egli Model [50]	Longley–Rice Model [51]	Okumura–Hata Model [52,53]
Terrain requirements	No strict constraint	Land: terrain data accuracy ≥1 km grid	Undulation height: <50 m Slope change: <15° Applicable terrain: irregular undulating terrain	Flat terrain: Δh < 30 m Moderate undulation: 30 m < Δh < 300 m Steep mountainous area: Δh > 300 m	Urban area: building heights are evenly distributed Suburbs: low- density buildings

**Table 5:** Key propagation parameters along the communication path.

Relative Permittivity	Conductivity
4.3	1.2 × 10^−3^

**Table 6:** Vehicle-mounted radio and antenna parameters.

Vehicle 1		Vehicle 2	
Working Condition	Transmitting	Working Condition	Receiving
Working frequency band (MHz)	30–80	Working frequency band (MHz)	30–88
Transmit power (W)	50	Sensitivity (dBm)	−107
Antenna height (m)	10 or 15	Antenna height (m)	12 or 15
Antenna gain (dB)	1	Antenna gain (dB)	1
Antenna polarization mode	Vertical polarization	Antenna polarization mode	Vertical polarization

**Table 7:** Radio wave propagation loss errors and comparison.

Working Frequency (MHz)	Transmitting Antenna Height (m)	Receiving Antenna Height (m)	Egli Model	Terrain- Corrected Egli Model	Optimized Model	Error Reduction	
						Compared to Egli Model	Compared to Terrain-Corrected Egli Model
40	10	12	9.64%	2.73%	0.46%	9.18%	2.27%
		15	9.70%	2.66%	0.48%	9.22%	2.12%
	15	12	9.73%	2.62%	0.38%	9.35%	2.24%
		15	9.77%	2.51%	0.45%	9.32%	2.06%
50	10	12	9.38%	2.57%	0.43%	8.95%	2.14%
		15	9.37%	2.46%	0.46%	8.91%	2%
	15	12	9.56%	2.55%	0.48%	9.08%	2.07%
		15	9.77%	2.64%	0.46%	9.31%	2.18%
70	10	12	9.26%	2.60%	0.45%	8.81%	2.15%
		15	9.21%	2.51%	0.34%	8.87%	2.17%
	15	12	9.35%	2.57%	0.38%	8.97%	2.19%
		15	9.43%	2.48%	0.41%	9.02%	2.07%
80	10	12	9.15%	2.55%	0.46%	8.69%	2.09%
		15	9.07%	2.29%	0.39%	8.68%	1.9%
	15	12	9.21%	2.44%	0.42%	8.79%	2.02%
		15	9.29%	2.41%	0.49%	8.8%	1.92%

## Data Availability

The complete dataset is subject to approval, but contour maps, topographic profiles, and some pieces of vehicle information are open source and sufficient to support the reproduction of this paper. Details can be found in “Construction and Analysis of the EMC Evaluation Model for Vehicular Communication Systems Based on Digital Maps” published in *Remote Sensing*.
